# An Address to the King and Parliament of Great Britain, on the Important Subject of Preserving the Lives of Its Inhabitants, by Means Which, with the Assistance of the Legislature, Would Be Rendered Simple, Clear, and Efficacious to the People at Large. With an Appendix, in Which Is Inserted a Letter from Dr. Lettsom to the Author

**Published:** 1782

**Authors:** 


X. *An addreſs to the King and Parliament of Great Britain, on the important ſubject of preſerving the lives of its inhabitants, by means which, with the aſſiſtance of the legiſlature, would be rendered ſimple, clear, and efficacious to the people at large. With an appendix, in which is inſerted a letter from* Dr. Lettſom *to the author. By* W. Hawes, M. D. *one of the inſtitutors of the Humane Society, Phyſician to the Surrey Diſpenſary, and reader of lectures on animation. To which are ſubjoined, hints for improving the art of reſtoring ſuſpended animation; and alſo for adminiſtering dephlogiſticated air in certain diſeaſes, and particularly in the preſent epidemic, termed Influenza, propoſed (in a letter to* Dr. Hawes) *by* A. Fothergill, M. D. *Member of the Royal College of Phyſicians, and* F.R.S. 8vo. Dodſley, *London*, 1782. 77 pages. ]

THE Humane Society has been eſtabliſhed only eight years, and it appears from the laſt report, that within that period 136 patients have been reſtored from apparent death. If

ſuch

ſuch has already been the ſucceſs of this new undertaking, under all the diſadvantages of popular prejudice, the ſcarcity of ſuitable apparatus, the diſtance oftentimes from ſkilful medical aid, and ſtill more the want of proper receiving houſes, much more might certainly be expected were theſe to be eſtabliſhed in their proper diſtricts, as in other countries, and the inſtitution made an object of the national police.—The addreſs to the King and Parliament, which extends through the firſt twenty pages of the work before us, is intended to draw the attention of the legiſlature to this important ſubject. Dr. Hawes propoſes that there be appointed by authority of Parliament, in every pariſh, one or more General Receiving-houſes, the expence to be defrayed by a general, county, or pariſh rate;—that at each of theſe places all the neceſſary apparatus, as proper medicines, an electrical machine, cupping inſtruments, a warm bath, beds, &c. be depoſited, and that a medical practitioner, with a moderate ſalary, and one or two other intelligent perſons properly qualified to aſſiſt in the buſineſs be conſtantly reſident in the houſe.

The letter from Dr. Lettſom, mentioned in the title page, contains only his approbation of the above plan, and is comprized in a dozen

 lines; lines; but that from Dr. Fothergill to Dr. Hawes, fills the laſt 41 pages of the work, and affords us many judicious hints for improving the art of reſtoring ſuſpended animation, &c.

Dr. Fothergill expreſſes a wiſh that a certain criterion between poſitive and apparent death, beſides that of putrefaction, may be ſoon diſcovered. The popular idea, he obſerves, that life quits the body in an aerial form, at the inſtant reſpiration ceaſes, has introduced dangerous error. He is of opinion, that the vital principle, like that of electricity (to which he thinks it bears ſtrong affinity) often remains in a dormant ſtate, without betraying any ſigns of its preſence, till it happens to be rouſed by the proper modes of excitation. Hence he conſiders the clay-cold-hand, the ſtiffneſs of the limbs, the dilatation of the pupil, the cadaverous countenance, and in ſome caſes, even putrefaction itſelf as equivocal ſigns of abſolute death. He ſpeaks of a peculiar *glaſſineſs* of the eye, when accompanied with coldneſs and flaccidity of the ſkin, as being one of the moſt certain criterions of poſitive death. Another ſign, likewiſe, which he thinks deſerves our particular attention is, when air, blown into the mouth, paſſes without interruption through the whole alimentary canal, as as this affords a ſtrong preſumptive proof that the internal ſphincters have loſt their irritability, and of courſe that life is totally exhauſted.

Among other means of reſtoring ſuſpended animation he recommends inflation of the lungs with dephlogiſticated air; and the uſe of electricity, beginning with the ſlighteſt ſhocks, and gradually proceeding to make the electrical circuit paſs more briſkly in different directions through the region of the heart, the lungs, and ſpinal marrow. He obſerves, that at that critical period when ſlight twitchings or gaſpings mark the firſt dawn of returning life, inſtead of *increaſing*, it will be prudent to *diminiſh* the electrical current, leſt the vital ſpark be again extinguiſhed.

In caſes where the natural heat of the body, in conſequence of remaining ſome time under water, or expoſure to extreme cold, is apparently extinguiſhed, he thinks it may be very neceſſary to apply artificial heat, and for this purpoſe recommends a ſweating chair or warm bath heated to 100°. On the other hand he obſerves, that perſons ſuffocated by the fumes of charcoal require to be expoſed to the cold air, and cold water, and are liable to relapſe if brought into a warm room; that it is found requiſite to rub the frozen with ſnow; and that dephlogiſticated air ſeems to be peculiarly adapted to the recovery of thoſe who are overcome by mephitic vapours, as electricity does to thoſe who are ſtruck by lightning. He recommends the injecting warm dephlogiſticated air into the inteſtines inſtead of the fumes of tobacco. He thinks that the ſickneſs and univerſal languor which theſe fumes uſually produce on other occaſions, when they penetrate beyond the valve of the colon, but ill ſuits with the idea of reſtoring animation. He queries whether the ſame objection may not be urged againſt the operation of emetics in theſe caſes.

In a poſtſcript to his letter, Dr. Fothergill recommends the uſe of dephlogiſticated air as a corrector of the impure air of courts of judicature, hoſpitals, priſons, &c. He obſerves that the Pennſylvanian ſtove, which already excels in throwing out warm ſteams of atmoſpheric air, might eaſily be adapted to the above intention. He likewiſe adviſes (on theoretical grounds only, as he candidly confeſſes) the uſe of dephlogiſticated air in the Influenza. He recommends, on the authority of Abbé Fontana, the breathing this ſpecies of air through lime water, in order to make it ſerve for reſpiration thirty times as long long as in the ordinary way. This proceſs, if it would ſtand the teſt of experiment, would certainly be a diſcovery of the very firſt magnitude; but unfortunately, it has been proved by Dr. Prieſtley (*Exp. and Obſ. relating to various branches of Nat. Phil.* Vol. 2.), that both the fact and the reaſoning urged in ſupport of it are void of foundation.

